# Postirradiation Osteosarcoma of the Maxilla: A Case Report and Current Review of Literature

**DOI:** 10.1155/2009/876138

**Published:** 2009-05-25

**Authors:** Imene Chabchoub, Olfa Gharbi, Sami Remadi, Sami Limem, Amel Trabelsi, Makrem Hochlef, Leila Ben Fatma, Amel Landolsi, Moncef Mokni, Chekib Kraiem, Slim Ben Ahmed

**Affiliations:** ^1^Department of Medical Oncology, University Hospital Farhat Hached, Sousse 4000, Tunisia; ^2^Department of Pathology, University of Medicine, Sousse, Leopard Senghor Street, Sousse 4000, Tunisia

## Abstract

*Background*. Radiation-induced sarcomas are well-known potential late sequelae of radiation therapy. They are of rare occurrence in jaw bones and are even rarer in the maxilla. *Case report*. We report a case of radiation-induced osteosarcoma involving the maxilla in a patient treated with radiotherapy for nasopharyngeal carcinoma 14 years ago. Despite neoadjuvant chemotherapy, surgical treatment could not be performed, and the patient received palliative chemotherapy. *Conclusions*. Radiation-induced osteosarcomas are aggressive and often elude early detection and timely intervention, rapidly leading to early demise of afflicted patients. Long-term patient follow-up and a high index of suspicion are crucial for timely intervention.

## 1. Background

Radiotherapy is the standard treatment for nasopharyngeal carcinoma (NPC) [1]. Unfortunately, it can produce undesirable complications after treatment. When radiotherapy is used with curative intent, the possibility of late complications in the irradiated tissues must be considered. The spectrum of malignant neoplasms secondary to radiation of head and neck tumors is wide. It includes skin, thyroid, and bone neoplasms. Because radiation fields for NPC may include the skull base as well as maxilla, mandible, and pterygoid bones, radiation-induced osteosarcoma (RIS) can arise from these sites as one of the late complications of radiotherapy. These tumors are uncommon but aggressive, occurring after a latency period of five years or more following radiotherapy. Histological proof of sarcoma is necessary to distinguish it from other radiotherapy changes such as osteonecrosis.

In view of the large number of nasopharyngeal carcinoma cases, it is not surprising that postirradiation maxillary, temporal, and sphenoid malignancies are now being reported. We present a 49-year-old woman with osteosarcoma of the maxilla occuring 14 years after radiotherapy for NPC with a review of literature.

## 2. Case Report

A 49-year-old woman, treated in 1992 for NPC (T3N1M0) with radiotherapy (70 Gy over 35 sessions with large lateral opposing faciocervical fields and complementary anterior facial field), presented fourteen years later with pain and swelling of the cheek. The swelling was increasing in size and advancing towards the eye. Examination showed an expansile mass in the right maxillary region with local paresthesia and proptosis of the right eye. The overlying skin appeared normal.

**Figure 1:**
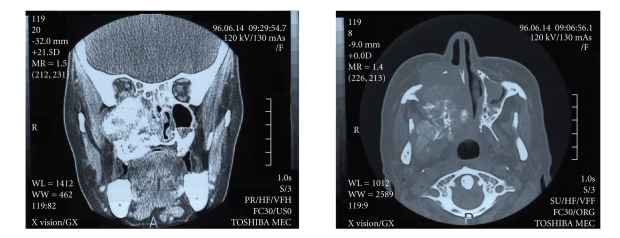
Bony mass in the left maxilla and extended to the right temporal bone.

**Figure 2:**
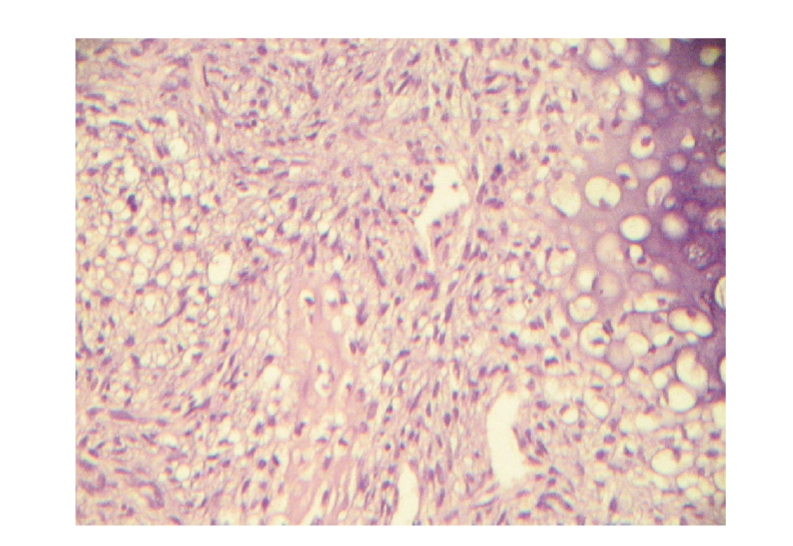
Section from the biopsy specimen showing a chondroblastic osteosarcoma which contains dominating chondroid substance (right side) and osteoid trabeculae derived from neoplastic cells (center) (He × 185).

Computed tomography (CT) demonstrated a large bony mass in the right maxilla that extended into temporal bone (Figure 1). Histological examination disclosed a fibroblastic osteosarcoma which produced chondroid substance, and contained distinct osteoid trabeculae. Immunohistochemistery showed diffuse positivity to vimentin and S100 protein antibodies (Figure 2). CT of the lung revealed no lung metastases.

After discussing the unresectability of the tumor with the patient, neoadjuvant chemotherapy was proposed with high-dose methotrexate. The tumor showed clinical and radiographic progression after 5 cycles of treatment. The mass extended into right orbit, right sphenoïd as well as the nasal cavity. A remedial treatment was started with doxorubicin and cisplatin, though CT appearances after chemotherapy were unchanged. Twelve months later, the patient is alive without metastases, and we are pursung palliative chemotherapy with ifosfamide and etoposide.

## 3. Discussion

The occurence of sarcomas in irradiated tissues is an uncommon but well-documented long-term complication of radiotherapy. The incidence of radiation-induced tumors is increasing in the oncology population as a result of increased survival, with improvement in cancer treatment [2–4]. In the largest study reported in English literature, Liu et al. [2] described 15 cases of RIS as a late complication of NPC. The study included 33.3% osteosarcoma of the maxilla, 46.7% of the mandible and 20% from a mixture of the nasal cavity and paranasal sinuses. They estimated the incidence of radiation-induced osteosarcoma in NPC as approximately 0.037% [2].

In 1948, Cahan et al. [5] described 11 cases of sarcomas arising from irradiated bones. They established four criteria for the diagnosis of radiation-induced osteosarcoma that are still valid today: (a) the origin of the neoplasm in the radiation field, (b) the nonmalignant nature of the initial bone condition (this criteria was modified by Arlen et al. [6] : “the tumors developed in bone not known to have a primary malignant osteoblastic lesion when the radiotherapy was given”), (c) the histological diagnosis of the neoplasm, and (d) a relatively long latency period. Our patient fulfilled the above-mentioned criteria.

The average standardized incidence of NPC in Tunisia in 1998 was 3.5 cases per 100 000 males and 1.6 cases per 100 000 females. More than 80 new cases of NPC are seen annually.

The most effective treatment is radiotherapy. The whole tumor dose delivered is generally 65 to 75 Gy. The dose targets the primary tumor and regional lymphatics, inclusive of the whole sphenoid body and posterior half of maxilla in all cases. Some other cases may need further intracavitary boost irradiation. In view of the large number of cases treated for this particular malignancy, it is not surprising that postirradiation sarcomas are now being reported in this population.

In general, a radiation dose of at least 30 Gy is required for the development of radiation-induced sarcoma [5, 7]. Reported radiation doses vary in this population from 25 to 110 Gy, with a median of 45 Gy [7]. Our patient received 70 Gy.

The latency period between radiotherapy for NPC and the development of temporal bone tumors ranged from 5 to 30 years with a mean of 12.9 years [8]. Our patient developed the second tumor 14 years after primary radiation therapy.

Although the pathogenesis is unknown, various predisposing factors have been suggested [9]. RIS may occur after megavoltage or orthovoltage radiation, but in the latter case, the treatment dose is lower and the period of irradiation is longer [10]. In addition to radiation dose, development of RIS is probably influenced by other factors: age of the patient at radiation exposure, association of chemotherapy, and genetic predisposition [11].

It is suggested that the patients who harbor mutations in tumor suppressor genes like *p53* and *RB1* are more prone to develop radiation-induced osteosarcoma. Friend et al. [12] described 8 cases of osteosarcoma with an altered RB allele. Others have suggested that the lack of RB expression in osteosarcoma and soft tissue sarcoma may be common since RB gene transcripts were not detected. Moreover, they consider that lack of of RB gene expression occurred only after the tumor develops. Nevertheless, radiation-induced DNA alteration is well demonstrated, so we could consider that deletion or inactivation of this tumor suppressor gene may be an important factor for RIS [12] with a relative risk of RIS 400-fold greater than the general population [13].

The Li-Fraumeni syndrome, von Recklinghausen's disease, and other hereditary syndromes have also been identified as risk factors for RIS [14]. In fact, radiotherapy delivered for benign diseases such as fibrous dysplasia or Paget's disease in the past may lead to RIS [15]. In fact, some studies have proved that RIS are increased after treatment of Hodgkin's lymphoma with radiotherapy. In addition, radiation therapy given during childhood can also increase the relative risk of RIS to 30 [11]. Leclercq et al. [11] have suggested also that addition of alkylator-based chemotherapy to radiotherapy will increase the risk of developing RIS threefold.

The most common clinical findings of the head and neck radiation-induced sarcoma are pain and swelling with pathological fracture also reported. A soft tissue mass and bone destruction due to new bone formation are the main characteristics on radiography and CT. The degree of new bone formation on CT is variable.

Chondroblastic osteosarcoma of the head and neck represents 25% of all osteosarcoma of this region with a low histologic grade encountered in 26% of cases [16]. The treatment of sarcoma of the maxilla irrespective of etiology includes radical surgery with adjuvant chemotherapy [16]. For the majority of authors, complete surgical excision with negative surgical margins is crucial to local control as well as recurrence-free survival. Some authors have recommended neoadjuvant chemotherapy before definitive surgery is undertaken [17]. The majority of authors in the reported cases and series use the same chemotherapy as used for primary sarcoma with ifosfamide, methorexate, and doxorubicin, though there are no randomised trials to compare results. Surgery appears to offer the best chance of cure. However, as osteosarcoma metastasizes by the hematogenous route, there is rationale for the addition of adjuvant chemotherapy [4]. There have not been large studies to determine the effectiveness of various treatments for postirradiation osteosarcoma.

Tumor size and grade are the two most important prognostic factors for soft tissue sarcomas, including those associated with radiation therapy. The treatment of choice for RIS is surgical resection with negative margins [18]. The role of adjuvant therapy in radiation-induced sarcomas is unknown. It seems that there is no survival benefit for those patients receiving adjuvant chemotherapy or radiotherapy compared with those treated only with surgery that achieves negative margins [11–18].

High-grade tumors that are larger than 5 cm should be treated with primary chemotherapy followed by complete surgical excision of residual disease. All low-grade tumors and high-grade tumors that are 5 cm or smaller should be treated with a margin-negative surgical excision, and systemic chemotherapy should be considered when a negative margin is not accomplished [17].

Osteosarcomas of the long bones frequently develop metastatic tumor, especially to the lungs. In contrast, RIS of the skull does not exhibit this tendency. Moreover, these tumors have a poor prognosis because of rapid local growth [19].

The prognosis of radiation-induced sarcoma is generally thought to be worse than primary sarcomas, regardless of site. The cumulative disease-free survival at 5 years for patients with a postirradiation osteosarcoma was 17%, with a median survival estimate of 1 year [14]. In a review of 78 cases from the Mayo Clinic, about 30% of the patients with sarcomas of the craniofacial bones survived 5 years without recurrence [20].

Most series report overall survival rates at 5 years in the range of 10% to 30% [14–21]. Several factors predict for this poor survival rate, including neurosensory symptoms at presentation, increasing patient age, and surgical margins less than 5 mm [7–11]. In multivariate analysis of 160 cases of RIS, Brady et al. [22] demonstrated that three variables had prognostic significance: the presence of metastatic disease, the completeness of surgical resection in patients with localized disease, and the size of the primary tumor in patients who underwent complete resection. Survival was independent of histologic subtype or site of disease.

Often, there is a delay in diagnosis until an advanced stage of disease because of the difficult distinction between neoplastic and radiation changes. In addition, RIS occurs often in anatomic sites (head and neck, pelvic, thorax) where complete surgical resection is difficult.

To prevent radiation-induced sarcoma, it is important to have meticulous attention to radiation dosimetry in carefully planned fields. For our case, the current radiation dose is less than in the past with the addition of chemotherapy in the treatment of NPC. Furthermore, intensity modulation radiotherapy (IMRT) will reduce the exposure of normal tissues to radiation.

Because of the aggressive nature of RIS, careful long-term follow-up of irradiated patients is crucial. A high index of suspicion is needed for early detection and timely intervention.

## 4. Conclusion

Radiation-induced sarcoma is a potential late sequelae of radiation therapy. These tumors are very aggressive and often elude early detection, thereby hindering timely intervention. Although early detection of radiation-induced tumors is important, it is difficult since changes seen after radiotherapy have often been attributed to other causes such as osteonecrosis. Although the condition is rare, the possibility of RIS must be considered in any patient who has received radiation treatment.
